# P4HA1 Regulates CD31 *via* COL6A1 in the Transition of Glioblastoma Stem-Like Cells to Tumor Endothelioid Cells

**DOI:** 10.3389/fonc.2022.836511

**Published:** 2022-04-13

**Authors:** Xiangming Han, Qiyan Wang, Sheng Fang, Jialin Wang, Fusheng Liu, Junwen Zhang, Guishan Jin

**Affiliations:** Brain Tumor Research Center, Beijing Neurosurgical Institute, Beijing Laboratory of Biomedical Materials, Beijing Tiantan Hospital Affiliated to Capital Medical University, Beijing, China

**Keywords:** P4HA1, COL6A1, CD31, transdifferentiation, glioblastoma stem-like cell, tumor endothelioid cells

## Abstract

Glioblastoma multiforme (GBM) is a common intracranial malignancy characterized by abundant and aberrant vasculature. The efficiency of existing antivascular treatments remains unsatisfactory. The transition of glioblastoma stem-like cells (GSCs) into tumor endothelioid cells (ECs) has been thought to cause glioma neovascularization and anti-angiogenesis tolerance, but the mechanisms regulating glioma transdifferentiation remains unclear. Our previous study found that P4HA1 regulates GSCs vascular mimicry in a hypoxic microenvironment, but the detailed molecular mechanism has not been determined. In this study, candidate protein COL6A1 was screened by mass spectrometry. *In vitro* experiments show that P4HA1 regulates the expression of CD31 *via* COL6A1, with the levels of expression of P4HA1, COL6A1 and the vascular endothelial molecular markers CD31 showing positive correlations *in vivo* assay. Altering the expression of P4HA1 in GSCs altered the expression of COL6A1 and CD31, thereby inducing glioma angiogenesis. In conclusion, this study revealed that the P4HA1/COL6A1 axis modulates the transdifferentiation process of GSCs into ECs. Interrupting this signaling axis can inhibit glioma angiogenesis, suggesting that this axis may be a novel target for antivascular therapy in patients with glioma.

## Introduction

Glioblastoma multiforme (GBM) is a highly vascularized malignant tumor characterized by microvascular hyperplasia (1). Despite significant advances in combined therapy, including surgery, radiotherapy and chemotherapy, the median overall survival in patients with GBM remains < 15 months, with the highest 5-year survival reported to date of 9.8% (2). Antivascular agents have therefore been used to treat patients with glioma; however, although anti-angiogenic agents have limited therapeutic effects in some patients, they induce some gliomas to become more invasive and aggressive (3, 4). Glioblastoma stem-like cells (GSCs) have been proved to differentiate into tumor endothelioid cells (ECs) to supply blood to tumors, resulting in the resistance of GBMs to anti-angiogenic treatment (5–7). Examination of ECs arising from GSCs may provide novel insights into the mechanisms underlying GBM neovascularization and suggest anti-angiogenic treatment targets (8).

Hypoxia microenvironment and hypoxia-inducible factor-1α (HIF-1a) pathway were regarded as intensive stimulators of the malignant biological progression of GBM, participating in the regulation of tumor cell proliferation, invasion, migration, and epithelial-mesenchymal transition (EMT), enabling neovascularization by enhancing GSCs differentiation into ECs (9, 10). HIF-1α has been demonstrated to directly regulate prolyl 4-hydroxylase subunit alpha 1 (P4HA1), a protein involved in cell metabolism under hypoxic conditions (11, 12). In addition, hypoxia activates collagen synthesis by upregulating hydroxylases P4HA1 and PLOD2 in a HIF-1α-dependent manner (13). P4HA1 was also shown to promote glioblastoma cell migration and invasion by facilitating the EMT process in hypoxic microenvironments (14) and to be an enzyme essential for vasifaction and maintenance of vascular wall integrity in malignancies (15, 16). Taken together, these findings connote that P4HA1 might link angiogenic processes in tumor cells to hypoxic microenvironments. However, the molecular mechanisms by which P4HA1 is involved in GBM angiogenesis and the specific role of P4HA1 in vascular mimicry of GSCs under hypoxic conditions remain obscure.

The level of expression of the endothelial cell markers CD31 has been described to correlate with glioma invasiveness and patient prognosis (17, 18). Moreover, platelet and endothelial cell adhesion molecule 1 (PECAM-1, CD31) was determined to promote endothelial cell migration by enhancing integrin-dependent adhesion to its ligand (19), which is crucial for vascular endothelial cell adhesion and stabilization of vascular integrity during vascularization (20).In this study, we revealed that P4HA1 significantly increased the level of CD31 by increasing the level of collagen type VI alpha 1 chain (COL6A1) protein in GSCs under hypoxic conditions *in vitro*. These findings suggested that P4HA1/COL6A1 signal axis can drive the vascular mimicry process of GSCs and encourage the expression of vascular endothelial markers CD31 in GSCs, thus favoring tumor angiogenesis and the malignant progression of glioblastoma. This novel signal axis can allow the transformation process of GSCs into ECs in response to the hypoxic microenvironment and generating new blood vessels, thus improving oxygen supply, which offers a promising target for antivascular therapy in patients with GBM.

## Materials and Methods

### Glioma Samples

The following brain tumor samples used for immunofluorescence were provided by the Department of Neurosurgery of Beijing Tiantan Hospital: 10 GBM, 10 grade III anaplastic astrocytomas, 10 grade II astrocytomas, and 2 grade I mixed neuronal-glial tumor samples. Tumors were histopathologically classified according to WHO classification. Informed consent was obtained from each patient, and the ethics review board of the Beijing Tiantan Hospital approved experiments (Ethical number: KYSB2017-004).

### Cell Culture

The human U87 and U251 glioma cell lines and the mouse GL261 glioma cell line were purchased from the Chinese Academy of Medical Sciences (Beijing, China) and cultured in Dulbecco’s modified Eagle medium (DMEM, Gibco, USA) containing 10% fetal bovine serum (FBS, Gibco, USA) in an incubator at 37°C with 5% CO_2_. The extraction and culture of GSCs refer to the methods described by Zhu et al. (21) and Zhang et al. (22). Dissociated U87, U251, and GL261 cells were washed twice with phosphate-buffered saline (PBS, pH 7.2) to eliminate FBS and then resuspended in serum-free DMEM/F-12 medium (Gibco, USA) supplemented with basic fibroblast growth factor (bFGF, 20 ng/ml, Peprotech), epidermal growth factor (EGF, 20 ng/ml, Peprotech, USA), and 2% B27 (Life Technologies Corporation, Grand Island, NY, USA). The medium was half replaced every 3 days and neurospheres were reseeded every 7 days after dissociation with Accutase (Gibco, USA).

### Lentivirus Infection

The lentiviral vectors were designed and synthesized by Genechem Shanghai Ltd. For transfection, single-cell suspensions of GSCs were divided into single cells and plated at a concentration of 5 x 10^3^ cells/well in 96-well plates. The cells were separately transfected with lentiviral vectors expressing P4HA1-eRFP (enhanced red fluorescence protein), eRFP alone (negative oeP4HA1 control group), shRNA targeting P4HA1, and scrambled nontargeting shRNA as shCtrl group in serum-free medium for 12 hours. Subsequently, the cells were washed and cultured in a serum-free medium for 3 days and lysed to extract proteins for western blot analysis of P4HA1 expression. In addition, GL261-derived GSCs were transfected with lentivirus expressing luciferase (Luc) and subsequently incubated with luciferase substrate for verification afterward to obtain *in vivo* imaging of tumor size in later experiments.

### Transfection of Small-Interfering RNAs

Control siRNA (sc-37007) and COL6A1 siRNA (sc-35085) were purchased from Santa Cruz Biotechnology (Beijing, China). A 2 ml aliquot of cells in serum-free medium was seeded at a concentration of 2 x 10^5^ cells/well to six-well tissue culture plates. Then cells were cultured at 37°C in a CO_2_ incubator for 2 days. The cells in each well were washed once with 2 ml of siRNA transfection medium (Santa Cruz, Beijing). The cells were gently resuspended, overlaid with siRNA in siRNA transfection medium, and incubated for 7 hours at 37°C, followed by the addition of 1 ml serum-free medium per well. The medium was replaced with a fresh serum-free medium after cells were incubated for 24 h. The assay was performed after cells were cultured for an additional 72 hours.

### Differentiation of GSCs into ECs

Single-cell suspensions of GSCs were cultivated in endothelial differentiation medium, consisting of DMEM/F12 supplemented with 2% FBS (STEMCELL Technologies Inc, Canada), 10 ng/ml VEGF (Invitrogen, USA), 1% B27 supplement, 10 ng/ml EGF, and 5 ng/ml bFGF, as described previously (10, 23–25). The cells were cultured for 72 hours at 37°C in a humidified incubator with an atmosphere consisting of 1% O_2_, 5% CO_2,_ and 94% N_2_. Cell appearance was monitored, followed by immunofluorescence and western blotting with the anti-CD31 antibodies to ensure that the transdifferentiation process had occurred.

### Tube Formation Assay

Each well of a 24-well plate was coated with 0.2 mL matrigel matrix (BD Biosciences) and allowed to solidify at 37°C for 30 min. GSCs were dissociated into single cells, resuspended at 1 x 10^4^ cells/mL in an endothelial differentiation medium containing 2% FBS, and incubated at 37°C in a humidified incubator with 1% O_2_ and 5% CO_2_ for 24 hours.

### Western Blotting

Cells were lysed in NP40 lysis buffer, and proteins were electrophoresed on 10% SDS-PAGE gels in Tris-glycine buffer and transferred to nitrocellulose filter membranes. Non-specific protein binding was inhibited by incubating the membranes in a solution of non-fat milk powder. The membranes were incubated overnight at 4°C with primary antibodies against P4HA1 (1:1000; Proteintech), CD31 (1:1000; Cell Signaling Technology), CD34 (1:1000; Abcam), and β-actin (1:5000; Cell Signaling Technology). The blots were washed with Tris-HCl buffered saline containing 0.1% Tween 20 (TBST) and incubated with horseradish peroxidase-conjugated secondary antibodies. After washing with TBST, the protein bands were visualized by enhanced chemiluminescence (Thermofisher, USA). All Western blot analyses were performed in triplicate. The blots were scanned using an EPSON scanner, and the bands were quantified by ImageJ software. The relative expression of each target protein was normalized to that of β-actin in the same sample.

### Liquid Chromatography-Mass Spectrometry

LC/MS/MS analysis was performed as described (26). Briefly, U251 GSCs were induced to differentiate into ECs for 3 days. Sample containing 200 μg protein were denatured, reduced, and alkylated according to the iTRAQ protocol (AB SCIEX company), followed by overnight digestion at 37°C with sequencing grade modified trypsin (Promega, Beijing, China) solution. iTRAQ labeling and strong cation exchange chromatography were performed as described (27). Samples fractionated by capillary high-performance liquid chromatography were analyzed using a Triple TOF 5600 system (ABSciex, USA) to detect differentially expressed proteins. The accuracy of the results was verified by subsequent western blotting.

### Immunofluorescent Staining

ECs derived from GSCs were incubated with antibodies against P4HA1, CD31, and COL6A1, followed by Alexa Fluor 488 and Alexa Fluor 594 conjugated secondary antibodies (Cell Signaling Technology, USA) to visualize the primary antibodies complexes. Nuclei were counterstained with 4’,6-diamidino-2-phenylindole (DAPI, ThermoFisher Scientific, USA).

Human and mouse glioma tissue samples were fixed in 4% formalin solution, embedded in paraffin, and cut into 4 um sections for tissue slice immunofluorescence. After dewaxing, the tissue sections were immersed in citric acid solution and heated in a microwave oven (Siemens, Germany) for antigen retrieval. The tissue sections were incubated overnight at 4°C with antibodies to P4HA1 (1:200, Abcam, Cambridge, UK), COL6A1 (1:50, Santa Cruz, USA) and CD31 (1:400, Abcam, Cambridge, UK), followed by Alexa Fluor 488, Alexa Fluor 594 and Alexa Fluor 594 conjugated secondary antibodies. DAPI solution (ThermoFisher Scientific, USA) labelled cell nuclei. Images were captured in black and white mode by the corresponding channel in the Zeiss orthographic microscope and then colored.

### Intracranial Xenograft Tumor Model

All animal procedures were approved by the Committee on the Ethics of Animal Experiments of Beijing Neurosurgical Institute (Ethical number: 201904009). Each experimental group consisted of six 6-week-old male C57BL/6 mice. Aliquots of 1.5 × 10^5^ GL261-derived GSCs-Luc stably overexpressing P4HA1-eRFP, eRFP alone, as well as GSCs with shRNA targeting P4HA1 and scrambled nontargeting shRNA were suspended in 5 µL PBS, and then were stereotactically injected into the right caudate nucleus (1.8 mm mediolateral, 0.8 mm anterior-posterior, and 3 mm dorso-ventral) of mice, as described previously (28). The mice were weighed every day in the first group, and their overall survival was recorded. In the second group, tumor growth was monitored by an *in vitro* imaging system (IVIS), then specimens were analyzed immunohistochemically. As for the endpoint, the mice were euthanized when neurological symptoms occurred.

### Bioinformatic Analysis

Expression of P4HA1 in normal and glioma tissues, prognostic analysis, and co-expression analysis of P4HA1 and vascular-related genes were analyzed in R software (version 3.6.1) based on data from TCGA and GTEx databases.

### Statistical Analysis

Results were presented as means and standard deviations, or as medians and ranges, as appropriate. Normally distributed data in two groups were compared by one-way analysis of variance (ANOVA), whereas two-way ANOVA compared customarily distributed data in three or more groups. Survival curves were generated using the Kaplan–Meier method and compared using log-rank tests. The relationships between P4HA1 and other angiogenesis-related genes expression were evaluated by Spearman correlation analysis. All statistical analyses were performed using GraphPad Prism 7 software, with P ≤ 0.05 considered statistically significant.

## Results

### Dysregulation of P4HA1 in Glioma and its Correlation With the Number of Blood Vessels

**Figure 1:**
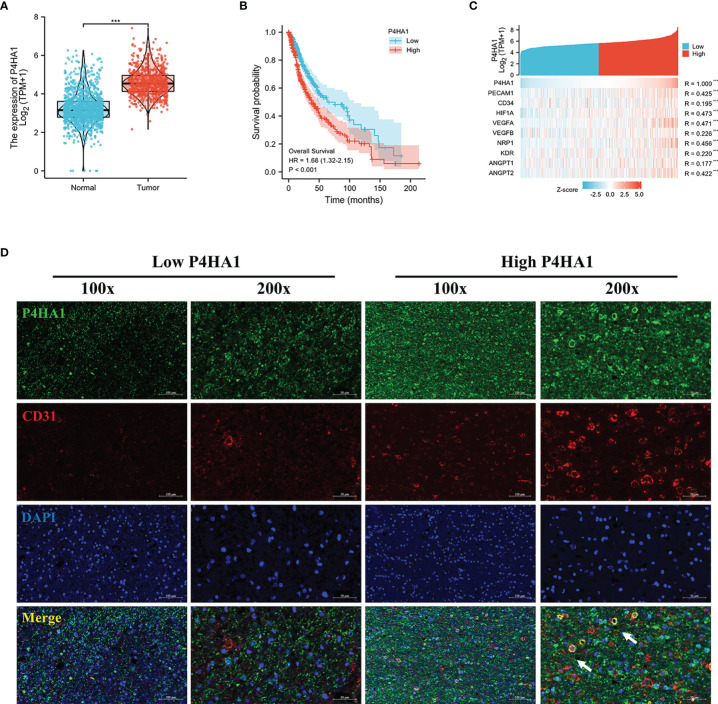
Dysregulation of P4HA1 in glioma and its correlation with the number of blood vessels. **(A)** Differential gene analysis of data from the TCGA and GTEx databases showed that P4HA1 expression was significantly higher in glioma tissues than in normal brain tissues. ***P < 0.001. **(B)** Overall survival analysis of patients with glioma from the TCGA indicated that patients with the high expression level of P4HA1 had a poor prognosis compared with those with the low expression level of P4HA1. **(C)** Spearman correlation analysis obtained the co-expression heat maps of P4HA1 expression and angiogenesis-related genes based on TCGA database. ***P < 0.001. **(D)** Immunofluorescent analysis of P4HA1 (green) and CD31 (red) showed that the expression of P4HA1 in clinical samples was correlated with the number of glioma blood vessels. The white arrow represents endothelial cells co-located with P4HA1 and CD31. 100X means that the magnification is 100 times(scale bar=100um), and 200X means 200X magnification(scale bar=50um).

Bioinformatic analysis results based on the TCGA and GTEx database (**Table S1**) revealed that *P4HA1* overexpressed in glioma compared with normal tissues (**Figure 1A**) and high expression of *P4HA1* correlated with poor overall survival (OS) (**Figure 1B**). Since P4HA1 was closely related to neovascularization (15), to investigate whether P4HA1 is concerned with vascularization in glioma, we analyzed the co-expression of P4HA1 and angiogenesis-related genes in the TCGA database. The TCGA database analysis showed that P4HA1 expression correlated positively with most vascular genes like *CD31*, *CD34*, *HIF-1a*, Vascular Endothelial Growth Factor A (*VEGFA*), vascular endothelial growth factor B (*VEGFB*), Vascular Endothelial Cell Growth Factor 165 Receptor (Neuropilin 1, *NRP1*), Vascular Endothelial Growth Factor Receptor 2 (Kinase Insert Domain Receptor, *KDR*) (**Figure 1C**). To verify whether P4HA1 is related to angiogenesis, immunofluorescence analysis was performed on P4HA1 and endothelial marker CD31 to evaluate the relationship between P4HA1 expression and blood vessels in patients’ specimens. Results showed that the expression level of CD31 was higher in glioma samples with high P4HA1 expression (**Figure 1D**), which connote that the expression of P4HA1 in glioma positively correlated with vascular density. Concurrently, we found that P4HA1 and CD31 were co-expressed in some vascular endothelial cells (indicated by white arrows in **Figure 1D**). Endothelial cell markers CD31 expressed in new and mature blood vessels exert substantial roles in sustaining stable endothelial cell connections (19, 20). As increment of blood vessels quantity in glioma denote pathological grade deterioration (29, 30), we speculated that P4HA1 might be involved in the process of glioma angiopoiesis in the malignant progression of glioma.

### P4HA1 Contributes to the Transition of GSCs-ECs

**Figure 2:**
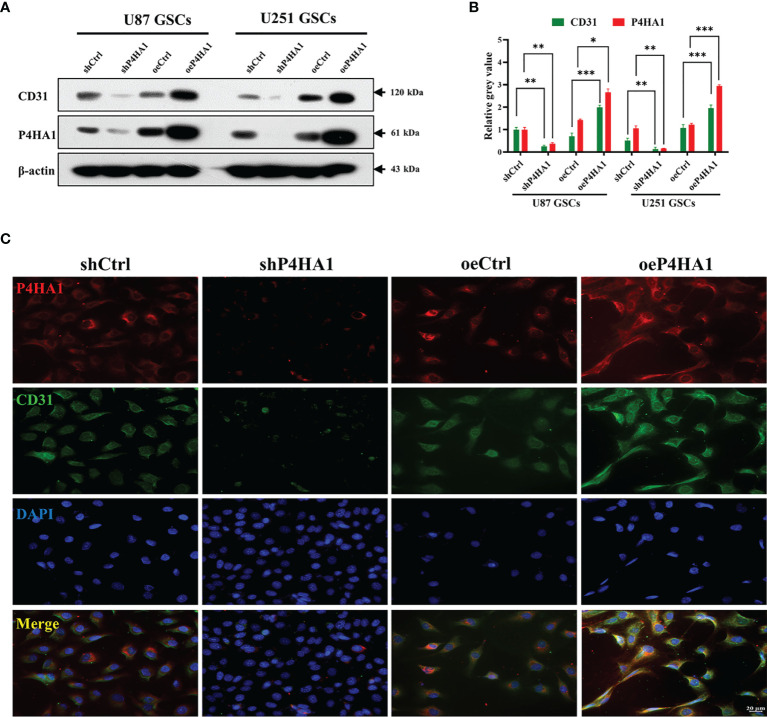
Transdifferentiation of GSCs with different P4HA1 expression levels. **(A)** Western blot analysis showing that CD31 expression was encouraged by the P4HA1 expression level in both U87 and U251 GSCs in the hypoxic transdifferentiation process. **(B)** Gray calculation of **(A)**. The gray value of each band was divided by the corresponding gray value of the actin band. All results were normalized to the gray value of the band of U87 GSCs transfected with shCtrl. *P < 0.05, **P < 0.01, ***P < 0.001. **(C)** Immunofluorescence assays showing that P4HA1 expression was positively correlated with CD31 expression in U251 GSCs after 72 hours hypoxic transdifferentiation (scale bar=20 μm).

Differentiation of GSCs into tumor endothelioid cells conduce to stable vascular structure in glioma, occupying an influential section in the vascularization of gliomas (5, 6). To verify whether P4HA1 can promote glioma angiogenesis, we induced GSCs to transdifferentiate *in vitro* to simulate angiogenesis *in vivo*. Firstly, Lentiviral vectors containing P4HA1-eRFP or eRFP were applied to infect GSCs derived from U87 and U251 cells and then conducted drug screening for 7 days, which constructed stable GSCs strain with P4HA1 overexpression or knockdown (**Figure S1**). The results of the western blot assay for detecting P4HA1 expression indicated that GSCs cell lines with different P4HA1 expression levels had been successfully constructed (**Figures 2A, B**). For the knockdown group, to ensure experimental correctness, two shRNA lentiviruses were adopted for knockdown followed by western blot, and the cell line with a better P4HA1 knockdown effect was selected (**Figure S2**). Then, GSCs were induced to undergo transdifferentiation in an endothelial cell differentiation medium. After 2 days of culture under hypoxia, the expression of P4HA1 and CD31 was detected by western blot assay. The results proved that knockout of P4HA1 significantly reduced the expression of endothelial cell marker CD31 in GSCs and overexpression of P4HA1 increased the expression of CD31 in GSCs by more than 40% (**Figures 2A, B**). Immunofluorescence experiments executed to scrutinize CD31 and P4HA1 expression yielded consistent results (**Figure 2C**). Therefore, these results represent P4HA1 capable of regulating the transdifferentiation of GSCs into ECs *in vitro*.

### P4HA1 Facilitates GSCs Tubular Vasculogenic Mimicry

**Figure 3:**
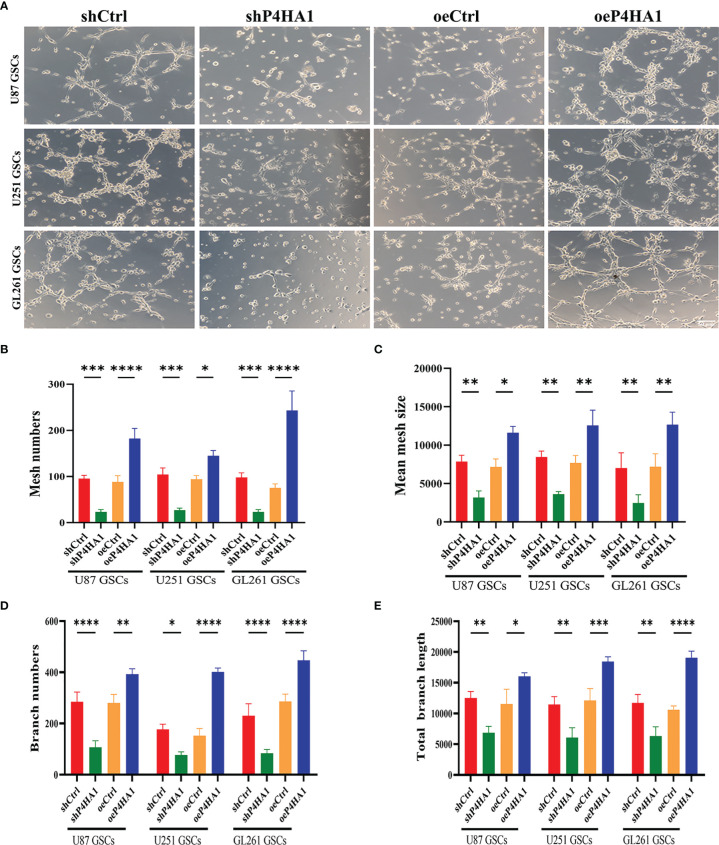
Tube formation ability of GSCs with different P4HA1 expression levels. **(A)** GSCs derived from U87, U251 and GL261 were incubated in endothelial cell culture medium and 1% O_2_ condition for 24 hours in a 24-well plate coated with 200ul matrigel matrix and then observed and photographed under a 200x light microscope(scale bar=50 μm). **(B–E)** Five visual fields were taken from each group, and the average mesh number **(B)**, mean mesh size **(C)**, the average branches number, and the average total branches length were calculated under ImageJ software. *P < 0.05, **P < 0.01, ***P < 0.001, ****P < 0.0001.

To further analyze the effect of P4HA1 on GSCs *in vitro* angiogenesis, we isolated U87, U251 and GL261 suspended neurospheres into single cells, resuspended them with endothelial cell culture medium, planted them in a 24-well plate coated with matrigel, incubated them in a hypoxic incubator for 48 hours, and then observed the formation of lumen under a microscope (**Figure 3A**). Since GSCs derived from GL261 were needed to construct *in-situ* mouse models later, GL261 GSCs were added for tumen formation analysis. The results showed that in oeP4HA1 group, the number of lumens (**Figure 3B**) was significantly higher, and the mean mesh size (**Figure 3C**) was more remarkable than that in oeCtrl group, the number of branches (**Figure 3D**) was also significantly higher and the total branch length (**Figure 3E**) was also longer than that in oeCtrl group. In shP4HA1 group, the mesh number, mesh mean size, branch number and total branch length were significantly decreased compared with the control group shCtrl. These results suggest that P4HA1 can effectively promote the angiogenesis of GSCs.

### P4HA1 Promotes COL6A1 Expression in GSCs Transdifferentiation

**Figure 4:**
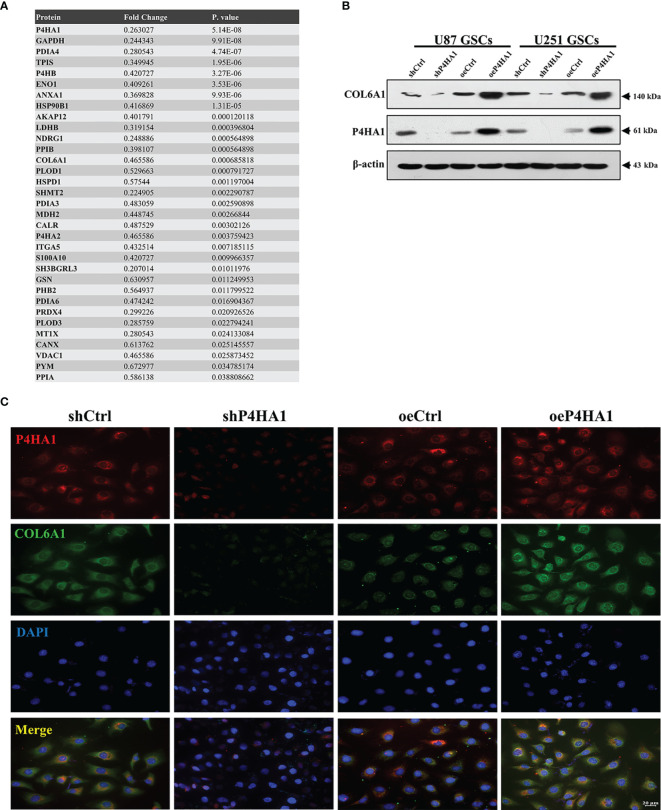
Candidate gene COL6A1 was screened as a downstream effector of P4HA1. **(A)** Liquid Chromatography-Mass Spectrometry results of U251 GSC-derived ECs transfected with shP4HA1 and shCtrl vector. Fold change was determined by the protein abundances. **(B)** Western blot analysis showing that heightened P4HA1 expression was associated with increased COL6A1 expression in both U87 and U251 GSCs under hypoxic transdifferentiation. **(C)** Immunofluorescence showing that P4HA1 expression was positively correlated with COL6A1 expression in U251 GSCs-derived ECs (scale bar=20 μm).

To determine the major downstream effector of P4HA1 driving the transdifferentiation process, LC-MS and western blot analysis of extracted and induced differentiated GSCs were implemented to obtain differentially expressed proteins related to P4HA1 expression. We screened this series of differentially expressed proteins, comprehensively analyzed the proteins whose molecular functions were related to angiogenesis and co-expressed with P4HA1 in glioma tissues, and finally identified COL6A1(**Figure 4A**). COL6A1 is a member of the collagen family (31). Recent evidence has proclaimed that COL6A1 was widely distributed in human malignant tumors, like prostate cancer, renal cell carcinoma, and cervical cancer (32–34), and involved in various biological functions of cancer cells, including cell migration, differentiation, and invasion. COL6A1 was uncovered to express characteristically higher in glioma than in surrounding normal tissues and extensively exist in the perivascular region (35). COL6A1, as a member of the collagen family, undertakes the main component of tumor ECMs (36), while P4HA1 can regulate the secretion of collagen, thus affecting the composition of ECM (37). These clues lead us to cogitate the internal relationship between P4HA1 and COL6A1. Hence, we embedded U87 and U251 GSCs with different P4HA1 expression levels into an endothelial cell medium under hypoxia conditions for 3 day and subsequently extracted proteins to run a western blot assay. Results conveyed that knockdown P4HA1 slashed COL6A1 protein levels compared with their respective controls, while overexpression of P4HA1 heightened COL6A1 protein levels (**Figure 4B**). In addition, immunofluorescence detection of U251 GSC-derived ECs transfected with overexpressing P4HA1 lentivirus showed that the expression levels of COL6A1 were raised with the increment of P4HA1 (**Figure 4C**), indicating that the expression of COL6A1 was regulated by P4HA1 during the differentiation process from GSC to ECs.

### Silence COL6A1 Suppresses the Transition of GSCs into ECs

**Figure 5:**
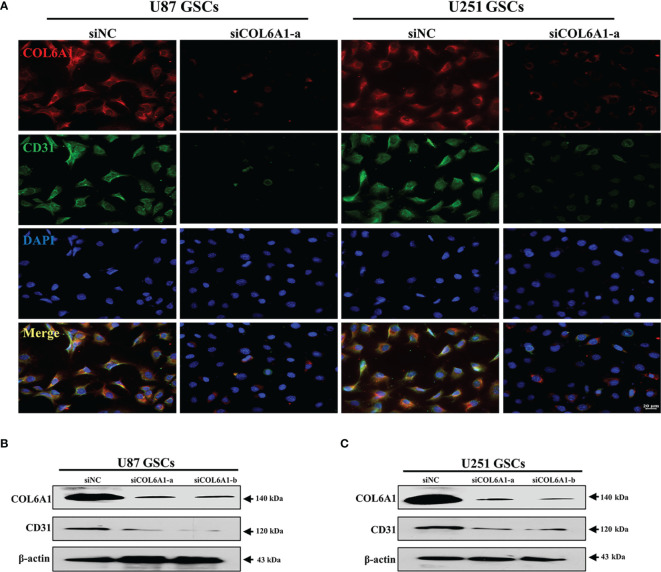
COL6A1 regulated GSCs differentiate toward ECs **(A)** Immunofluorescence analysis of COL6A1 and CD31 in U287 and U251 GSCs infected with siNC and siCOL6A1-a after GSCs induced transdifferentiation in hypoxia(scale bar=20 μm). **(B, C)** Western blot analysis of CD31 and COL6A1 in U87(B)or U251(C) GSCs transfected with siNC, siCOL6A1-a and siCOL6A1-b after 3 days of transdifferentiating induction.

Considering the regulatory relationship of P4HA1 on COL6A1 and the effect of P4HA1 on CD31 expression, we speculated that COL6A1 might also play a role in CD31 expression. Therefore, U87 and U251 GSCs were cultured in hypoxic conditions and differentiation medium for 3 days to induce differentiation into ECs after transfected the human GSCs with siRNA explicitly targeting the COL6A1. Then anti-COL6A1 antibodies were used to test the COL6A1 expression levels in U87 and U251-derived GSCs. As shown with the immunofluorescence result (**Figure 5A**), U87 and U251 GSCs-derived ECs were shown decreased CD31 levels with the condition COL6A1 expression level was downregulated compared to the control group. In addition, Western blot demonstrated CD31 expression was subsequently repressed after GSCs differentiation under hypoxia with the condition of COL6A1 expression reduced by siCOL6A1 (**Figures 5B, C**). These results indicated that COL6A1 is essential for the transition of GSCs into ECs

### Silence of COL6A1 Interrupts GSCs Transdifferentiation Enhanced by P4HA1 Under Hypoxia

**Figure 6:**
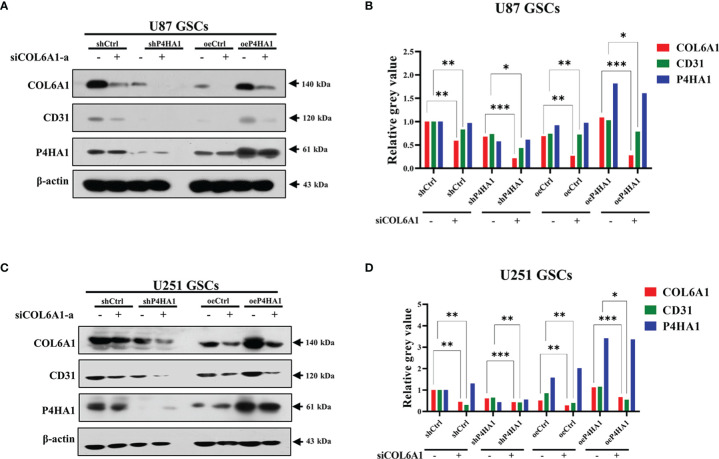
Silence of COL6A1 interrupts the promotion of P4HA1 in hypoxia-induced GSCs transdifferentiation. **(A, C)** U87 GSCs **(A)** or U251 **(C)** GSCs knocked down (sh P4HA1) or overexpressing P4HA1 (oeP4HA1) were transfected with siRNA interfering with control (siNC) or COL6A1 (siCOL6A1-a). 3 days of hypoxia induction was performed after transfection and cells were then collected, lysed and subjected to SDS-PAGE. Western assays were performed to detect expression of CD31, COL6A1, P4HA1 and β-actin with the indicated antibodies. **(B, D)** Gray calculation of A(B) or C(D). The gray value of each band was divided by the corresponding gray value of the actin band. All results were normalized to the grey value of the band of U87 GSC-derived or U251 GSC-derived ECs transfected with the shCtrl group. *P < 0.05, **P < 0.01, ***P < 0.001.

Previous findings showed that P4HA1 regulates COL6A1, and COL6A1 regulates the expression of CD31. To verify that P4HA1 regulates CD31 expression through COL6A1, we interfered with COL6A1 expression using siRNA in U87 (**Figures 6A, B**) and U251 (**Figures 6C, D**) GSCs that knocked down or overexpressed P4HA1, respectively. After transfection of siRNA, we checked the expression of P4HA1, COL6A1 and CD31. The results demonstrated that the expression of P4HA1 was not affected by knockdown of COL6A1 in U87 and U251 GSCs while the expression of CD31 is significantly suppressed. In P4HA1 overexpressing GSCs, both COL6A1 and CD31 were significantly increased, while CD31 expression was significantly inhibited after knockdown of COL6A1 despite high expression of P4HA1. These results suggest that the silencing of COL6A1 interrupts the promotion of P4HA1 during the transdifferentiation of GSCs into ECs.

### P4HA1 Promotes Glioma Progression and Expression of COL6A1 and CD31 *In Vivo*

**Figure 7:**
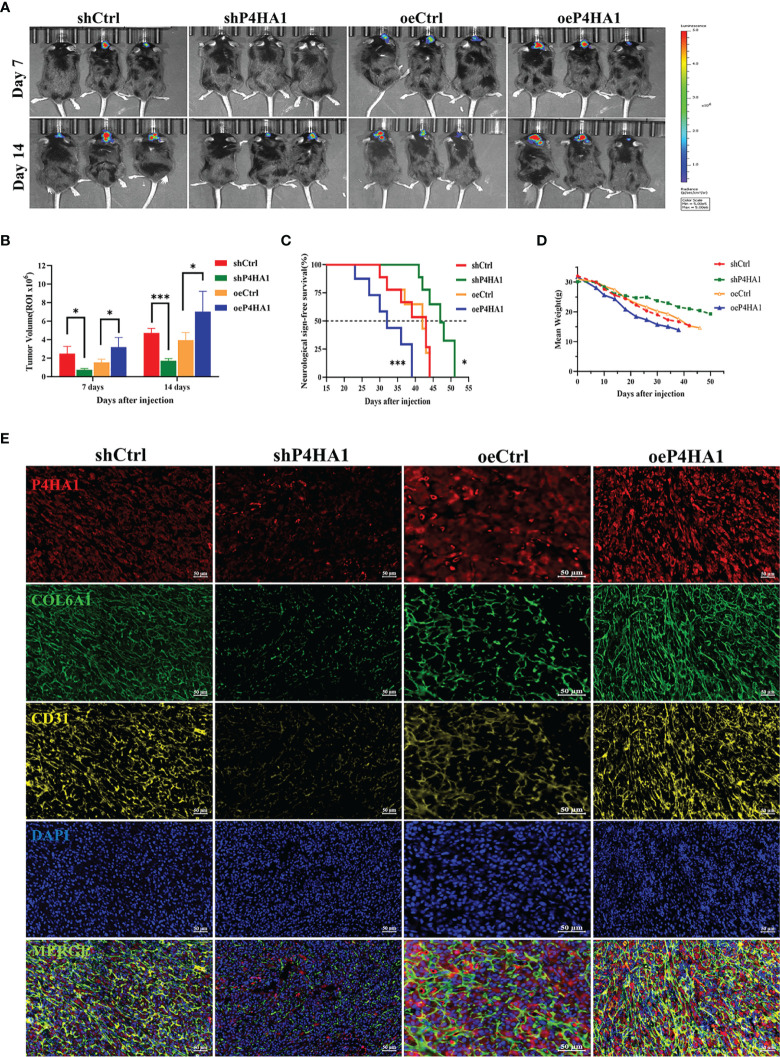
P4HA1 promotes glioma progression and expression of COL6A1 and CD31 *in vivo.* **(A)** *In vivo* imaging shows glioma growth 7 days and 14 days after injection of 1.5x10^5^ GL261 GSCs-Luc with different levels of P4HA1 into each mouse brain. **(B)** Intracranial tumors size calculation of **(A)**. *P < 0.05, ***P < 0.001. **(C)** Kaplan-Meier survival curve of mice implanted with GSCs transduced with shCtrl, shP4HA1, oeCtrl, oeP4HA1 lentiviral vector (n = 6 mice for per group). Log-rank test, *P < 0.05, ***P < 0.001. **(D)** Mice weight loss curve within 50 days post-implantation. **(E)** Polychromatic tissue immunofluorescence immunoassay in mouse tissue sections showed that the expression of P4HA1, COL6A1 and CD31 in mouse glioma was positively correlated and significantly co-located (scale =50 um).

To determine if similar effects are observed when cells are growing *in vivo*, GL261-derived-GSCs-Luc were implanted into the brains of C57BL/6 mice (15,000 cells/mouse), with tumors evaluated beginning 7 days later by the IVIS. GSCs transfected with lentivirus overexpressing P4HA1 yielded larger-sized gliomas than their controls after 7 and 14 d, whereas GSCs transfected with lentivirus shP4HA1 yielded smaller tumors than their controls (**Figures 7A, B**). In addition, tumors overexpressing P4HA1 grew significantly faster than tumors overexpressing vector alone (**Figure 7B**). The median overall survival (OS) time of the oeP4HA1 and oeCtrl group of animals was 32 and 42 days, respectively (**Figure 7C**). Meanwhile, the median OS times of mice implanted with GSCs expressing shP4HA1 and control shRNA were 43 and 47 days, with the later difference being statistically significant. After intracranial implantation of SCs in mice, the average body weight of each group was detected. According to linear regression analysis, the weight loss rate of the four groups was -0.4122(shCtrl), -0.2182(shP4HA1), -0.3831(oeCtrl), -0.4840(oeP4HA1) g/day, respectively, which indicated that P4HA1 knockdown slowed down the rate of weight loss in mice, while overexpression of P4HA1 accelerated it (**Figure 7D**). These results were consistent with the *in vitro* findings and suggested that P4HA1 could accelerate glioma growth and progression. Immunofluorescent analyses of tissue samples sections from these mice revealed the quantity of P4HA1, CD31, and COL6A1 was correlated positively in the four groups of tumors (**Figure 7E**), which tallied with the results of *in vitro* experiments before. These results showed that P4HA1 overexpression promoted angiogenesis and glioma growth *in vivo*.

## Discussion

GSCs are a small subset of glioma cells with stem cell characteristics and are widely believed to be responsible for poor prognosis in patients with glioma (38). GSCs are localized to and maintained in the glioma niche and are involved in glioblastoma vascularization and resistance to treatment (21, 39, 40).GSCs may undergo transdifferentiation into ECs, enhancing tumor resistance to currently available anti-VEGF agents (1). For example, temozolomide (TMZ) has been found to enhance the expression of both GSC and EC markers, leading to the generation of glioma-associated ECs and tumor-derived blood vessels (41, 42). Determining the mechanisms by which GSCs induce angiogenesis in the glioma microenvironment may identify new targets and treatment strategies for patients with glioma.

Hypoxic microenvironments have been proved to contribute to stimulating GSCs to adapt and differentiate into endothelial cells that supply blood to the tumor (9, 10, 43). P4HA1 is highly expressed in the hypoxic microenvironment, participating in a mutual regulatory feedback loop with HIF-1α (11, 12). Knockdown of P4HA1 inhibits neovascularization under hypoxic conditions and disrupts the vascular basement membrane (15), suggesting a relationship between P4HA1 and angiogenesis in glioma. Analysis of the TCGA database showed that P4HA1 was more highly expressed in glioma than in normal tissues. Moreover, Spearman’s analysis showed a positive correlation between P4HA1 and CD31, an endothelial marker that contributes to endothelial cell-cell junctions and the stability of blood vessels (18–20, 44). Furthermore, examination of human glioma specimens revealed a positive correlation between P4HA1 expression and blood vessel density, with endothelial cells in blood vessels co-located with P4HA1 and CD31 (**Figure 1**). To clarify the relationship between P4HA1 and vascular-related factor CD31, GSCs were induced to transdifferentiate toward ECs *in vitro*, simulating the angiogenic activity of stem cells *in vivo*. P4HA1 was found to enhance the transdifferentiation of GSCs to endothelioid cells and tube formation. In particular, overexpression of P4HA1 markedly enhanced CD31 molecular expression in GSCs derived ECs. Consistently, this finding was also observed that P4HA1 upregulated the level of classical vascular factors CD31 in the mouse glioma model (**Figure 7**).

To deep excavate the mechanism of action of P4HA1, proteins differentially expressed by GSCs that did and did not knock down P4HA1 were adopted by LC-MS. One of the molecules identified, COL6A1, a member of the collagen’s family, intrigued us. Collagens are one of the major protein constituents in the ECM, among which collagen VI plays an important role in interacting with a range of ECM components (45). P4HA1 has also been confirmed to be linked to hypoxia-induced ECM remodeling, local invasion, and angiogenesis (15, 46), so we suspected that P4HA1 and COL6A1 might be related in GSCdECs and the verification was also reflected in follow-up experiments. The co-immunoprecipitation assay revealed the physical interaction between P4HA1 and COL6A1 in U251 GSCs after 72 hours of hypoxic induction (**Figure S3**). And compared with their respective controls, shP4HA1 reduced COL6A1 protein expression and P4HA1 overexpression enhanced COL6A1 protein expression (**Figure 4B**). Similarly, oeP4HA1 group immunofluorescence in U251 GSCdECs also reflected the expressions of COL6A1 were raised as the increment of P4HA1 (**Figure 4C**), and immunofluorescence analysis of mouse tumor samples showed that the COL6A1 expression was controlled by P4HA1(**Figure 7**), which was consistent with the experimental results *in vitro*. This indicated COL6A1 might serve as a downstream molecule of P4HA1.

**Figure 8:**
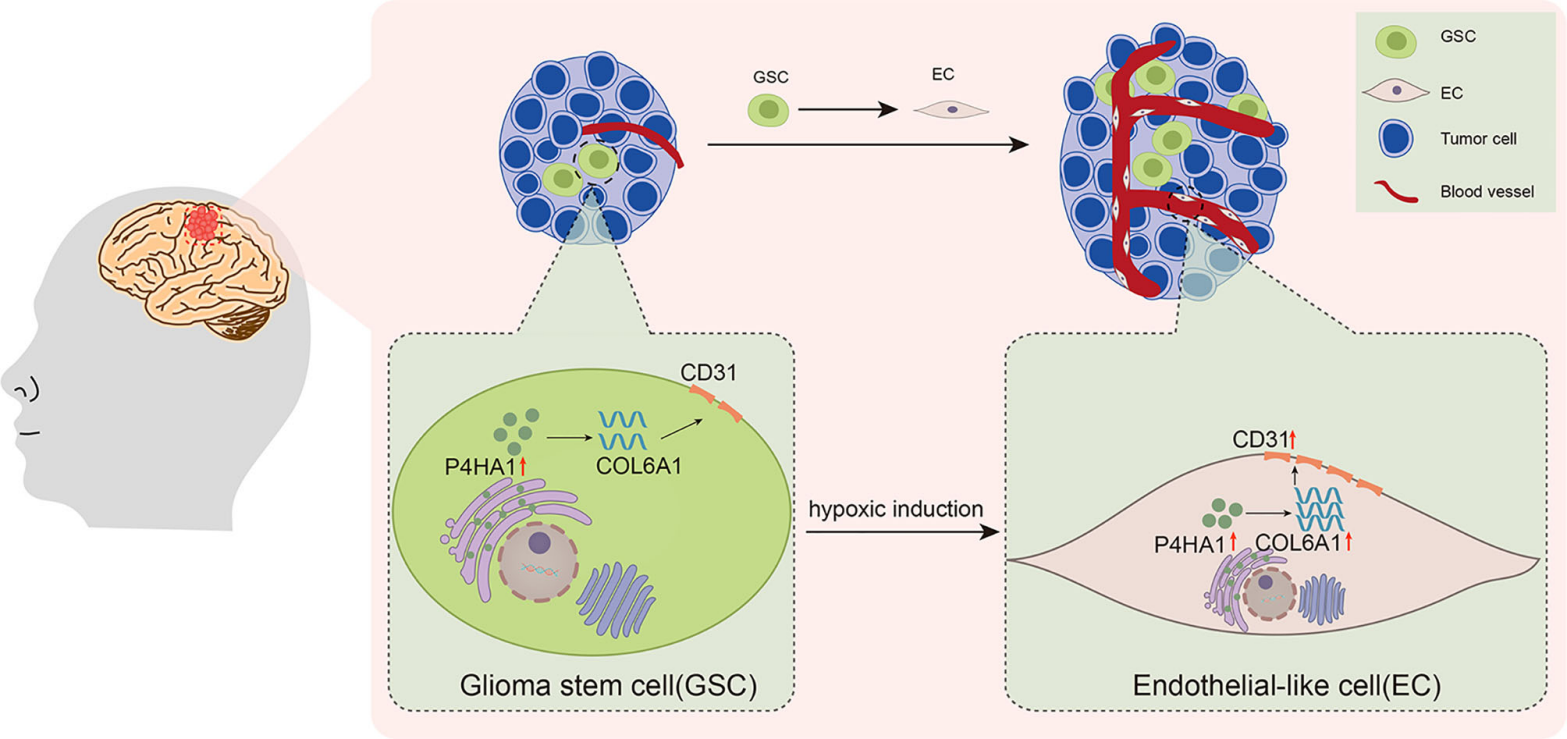
Schematic illustration of the activities of P4HA1, COL6A1, and CD31 in the process of GSCs transdifferentiation into ECs in glioma.

Recently, COL6A1 has been reported to influence clinical outcomes in patients with glioma, with increased expression levels, especially in GSCs (18, 35). Although COL6A1 has been studied to promote tumor progression and metastasis in other types of tumors, its specific function of COL6A1 has never been clarified in glioma. Thus, our findings of this study provide new clues for exploring the role of COL6A1 in glioma angiogenesis and progression. COL6A1 expression is generally limited to the perivascular region in glioblastomas but has also been observed in glioma cells that are organized in pseudopalisades (46), a familiar morphologic feature that links hypoxia, vascular pathology, and angiogenesis in glioblastoma (47). Glioma cells that form pseudopalisades are typical of high-grade glioma and are increasingly found around necrotic hotspots (47). The association of COL6A1 function with angiogenesis under a hypoxic microenvironment is supported by results showing that COL6A1 is overexpressed in the cerebral cortex after hypoxic-ischemic brain injury (48). COL6A1 may also be induced in normal brains after an injury resulting in reduced oxygen supply. Notably, the present study showed that COL6A1 facilitated the expression by GSCs of CD31 and their differentiation to ECs *in vitro* under hypoxic differentiation conditions (**Figure 5**). And after 3 d of hypoxic induction, silencing of COL6A1 reduced the expression of CD31 in GSC-derived ECs regardless of the overexpression of P4HA1, suggesting that COL6A1 controlled the expression of CD31 in these cells independently of P4HA1, though we set up groups with different P4HA1 expression levels. Interestingly, P4HA1 had been verified to control the expression of both CD31(**Figure 2**) and COL6A1 (**Figure 4**) in GSCs cultured under hypoxic conditions and *in vivo* (**Figure 7**). Given that transition of GSCs to ECs occurred in the hypoxic microenvironment (49, 50) and the upregulated expression of P4HA1 and COL6A1 under hypoxia (11, 51), combined with our experimental results, there might exist a PH4A1/COL6A1 regulation axis as an adaptive response to the hypoxic microenvironment, cooperating with the ECM to drive GSCs to differentiate into ECs and subsequently promote neovascularization in GBM (**Figure 8**). Our experiments demonstrated that this axis could promote the expression of endothelial cell marker CD31 in GSCs, thus reinforcing the interconnectivity between endothelial cells and stabilizing vascular structure. The study also has some limitations on whether P4HA1 and COL6A1 can promote angiogenesis in other type tumors like glioma remains to be defined.

In conclusion, our study revealed a novel mechanism in which the P4HA1/COL6A1 axis drives GSCs to transdifferentiate into ECs under hypoxic conditions. These findings may expand understanding of the mechanisms involved in glioma angiogenesis and provide a novel strategy for improving glioma treatment.

## Data Availability Statement

The original contributions presented in the study are included in the article/**Supplementary Material**. Further inquiries can be directed to the corresponding authors.

## Ethics Statement

The studies involving human participants were reviewed and approved by Beijing Tiantan Hospital Ethics Committee (Ethical number: KYSB2017-004). The patients/participants provided their written informed consent to participate in this study. The animal study was reviewed and approved by Beijing Neurosurgical Institute Ethics Committee (Ethical number: 201904009).

## Author Contributions

XH conducted data curation, investigation, resources, and writing original draft preparation. QW processed software and review. SF and JW performed the instrument and data analysis. FL managed funding acquisition and supervision. JZ and GJ contributed to funding acquisition, conceptualization, methodology, and supervision. All authors contributed to the article and approved the submitted version

## Funding

This work was supported by the National Natural Science Foundation of China (No. 81772671 and 81972344), the Natural Science Foundation of Beijing Municipality (No. 7202020), and the Beijing Laboratory of Biomedical Materials Foundation.

## Conflict of Interest

The authors declare that the research was conducted in the absence of any commercial or financial relationships that could be construed as a potential conflict of interest.

## Publisher’s Note

All claims expressed in this article are solely those of the authors and do not necessarily represent those of their affiliated organizations, or those of the publisher, the editors and the reviewers. Any product that may be evaluated in this article, or claim that may be made by its manufacturer, is not guaranteed or endorsed by the publisher.
