# Supplementary material for: P4HA1 Regulates CD31 via COL6A1 in the Transition of Glioblastoma Stem-Like Cells to Tumor Endothelioid Cells
Source: Front Oncol. 2022 Apr 13;12:836511. doi: 10.3389/fonc.2022.836511 (PMC9044633; doi:10.3389/fonc.2022.836511)
Supplement: Supplementary file 1 [file DataSheet_1.docx]

Figure 1B

Available in online tool Gepia(<http://gepia.cancer-pku.cn/detail.php?gene=&clicktag=boxplot>)

1.choose Expression DIY - Box Plots module,

2.set gene P4HA1, |Log2FC| Cutoff: 1, p-value Cutoff:0.01,

3.select GBM and LGG datasets, Log Scale:yes, Jitter Size:0.4, Matched Normal data: Match TCGA normal and GTEx data

4.click plot button

Figure 1C

Available in online tool Gepia(<http://gepia.cancer-pku.cn/detail.php>)

1.choose Correlation module,

2.set gene A: P4HA1, set gene B: PECAM1,

3.choose Spearman correlation coefficient analysis

3.select GBM tumor and LGG tumor datasets

4.click plot button

Figure 1D

Available in online tool Gepia(<http://gepia.cancer-pku.cn/detail.php>)

1.choose Correlation module,

2.set gene A: P4HA1, set gene B: CD34,

3.choose Spearman correlation coefficient analysis

3.select GBM tumor and LGG tumor datasets

4.click plot button

Figure 2A

Western blot CD31/P4HA1/β-actin in U87/251 GSCdEC


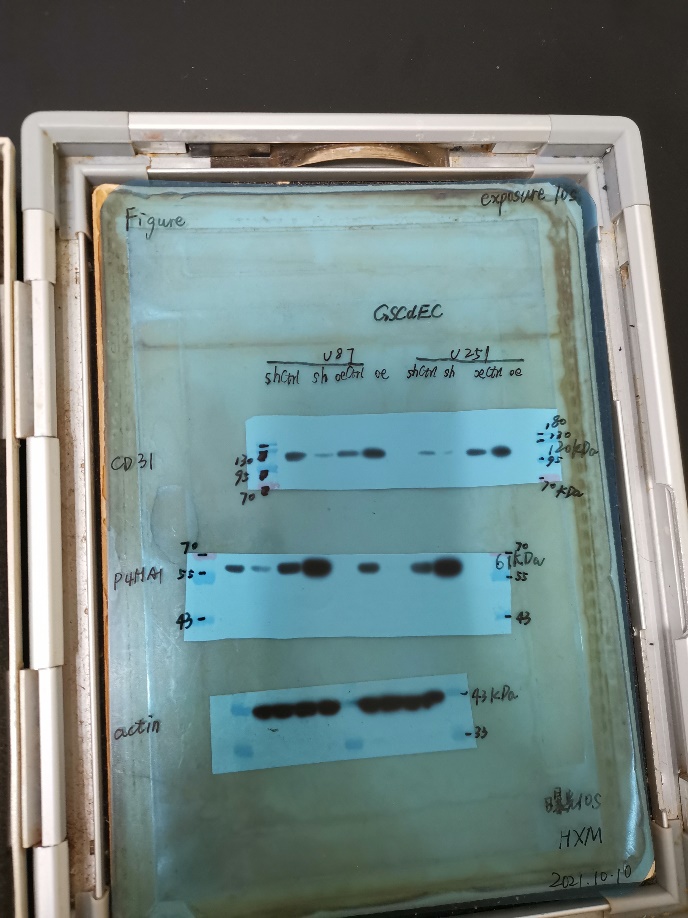


Figure 3A

LC-MS original source data was shown in another file named ***LC-MS original source data***

Figure 3B

Available in online tool Gepia(http://gepia.cancer-pku.cn/detail.php)

1.choose Correlation module,

2.set gene A: P4HA1, set gene B: COL6A1,

3.choose spearman correlation coefficient analysis

3.select GBM tumor and LGG tumor

4.click plot button

Figure 3C

Available in online database CGGA (http://www.cgga.org.cn/index.jsp)

1.choose Analyze - mRNA data- Distribution module,

2.input gene: COL6A1,

3.click submit button

Figure 3D

Available in online tool Gepia(http://gepia.cancer-pku.cn/detail.php)

1.choose Survival-survival plot module,

2.input gene: COL6A1,

3.choose overall survival, group cutoff: Quartile

4. set Hazards Ratio (HR): Yes, set 95% Confidence Interval: Yes, set Axis Units: months

5.select GBM tumor and LGG tumor

6.click plot button

Figure 4A


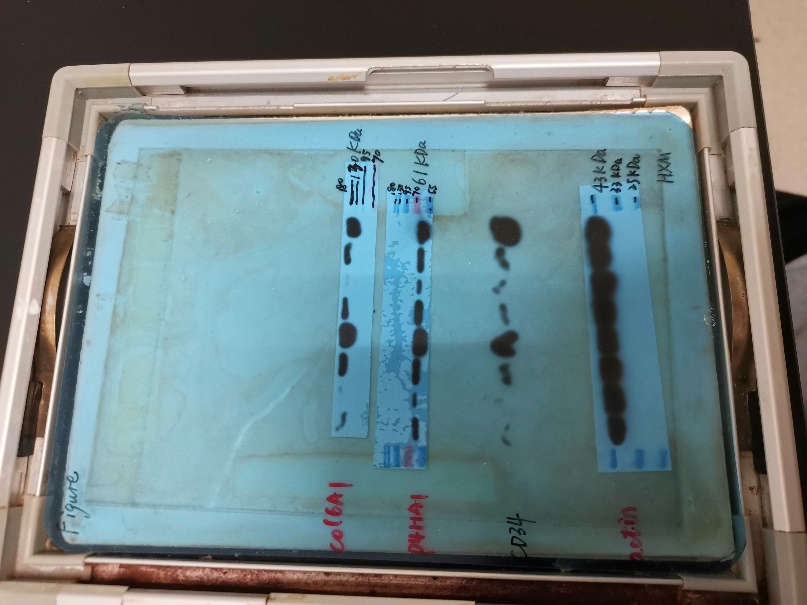

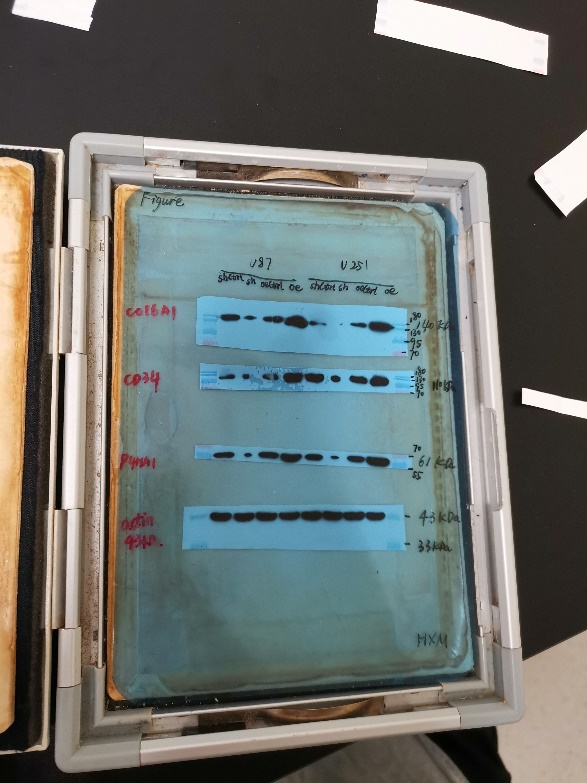


Figure 5B


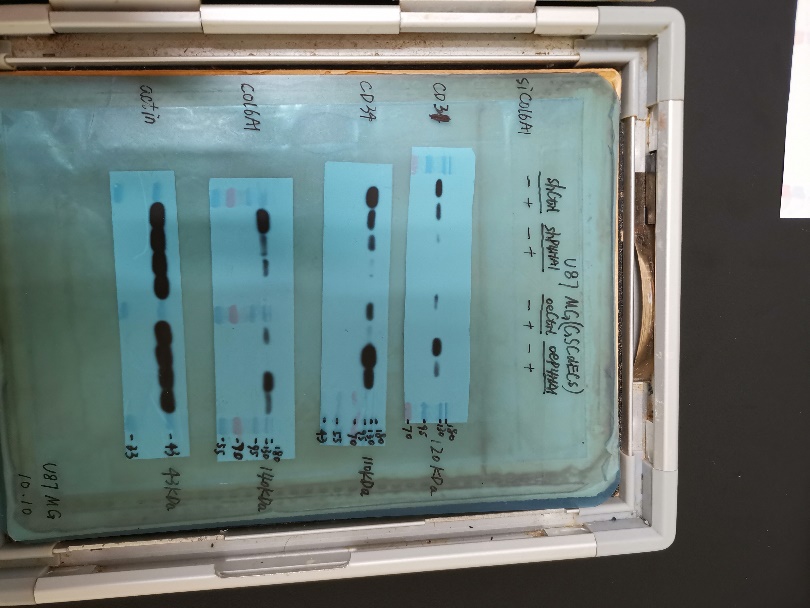

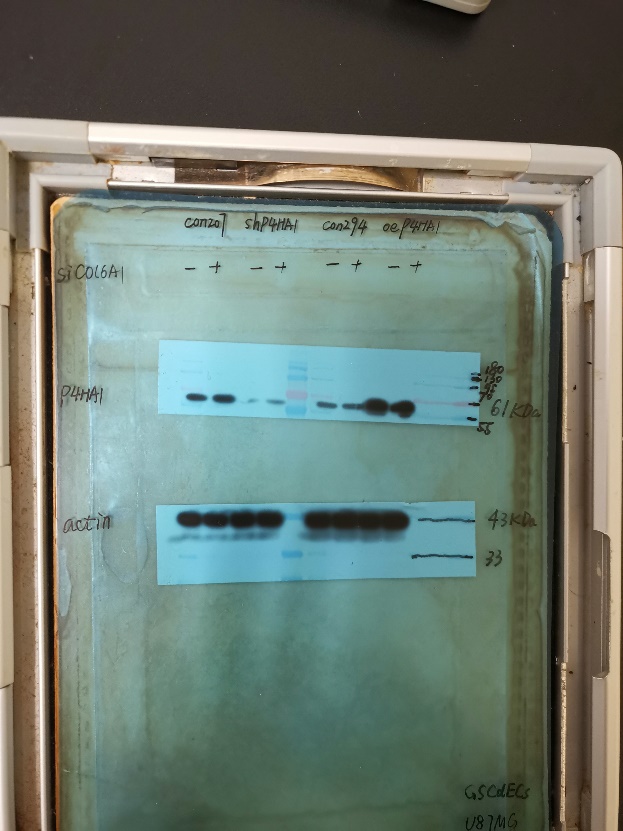


Figure 5D


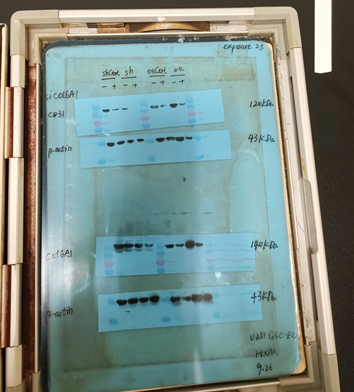

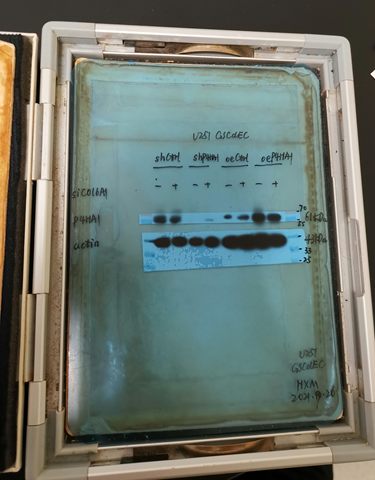


Figure 6A


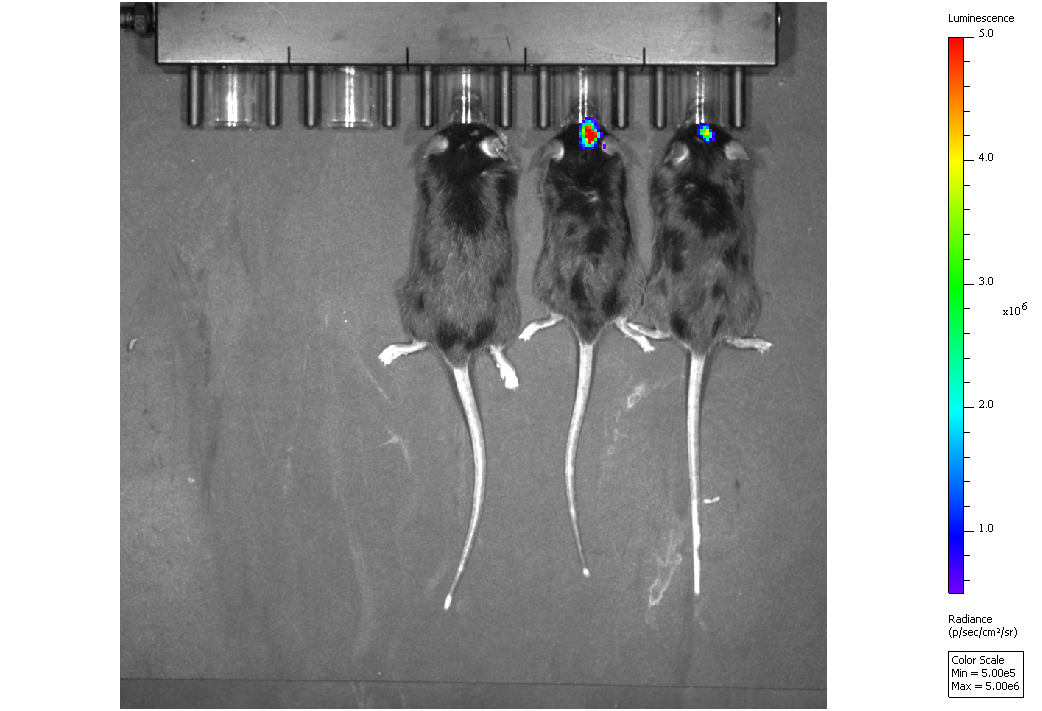
shCtrl(day7)
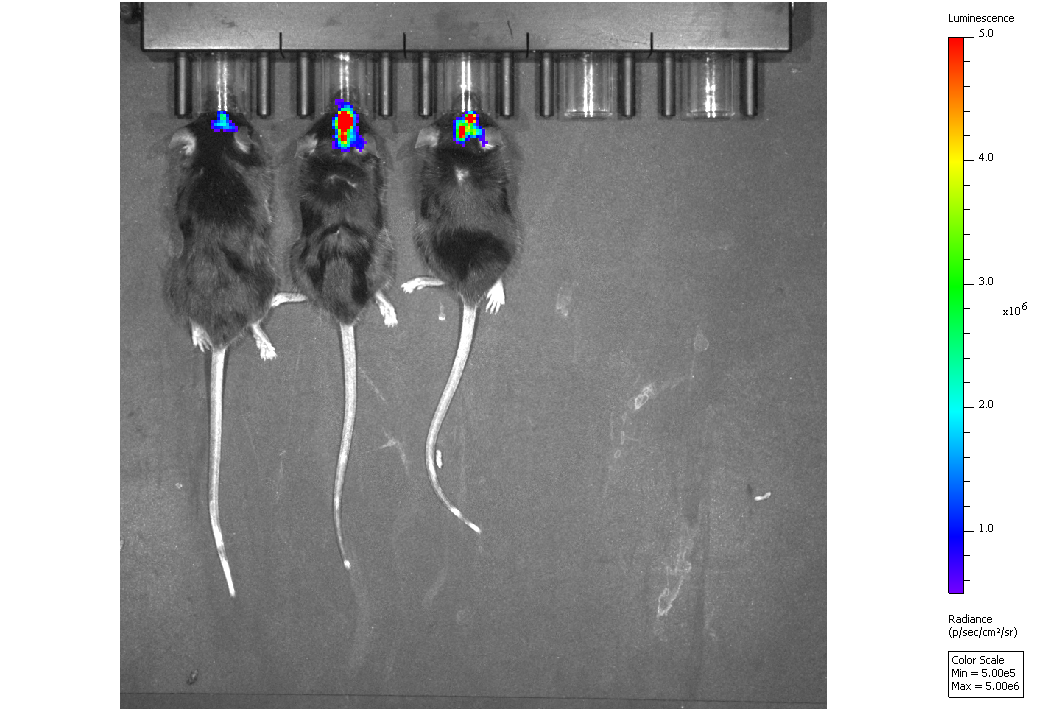
shCtrl(day14)


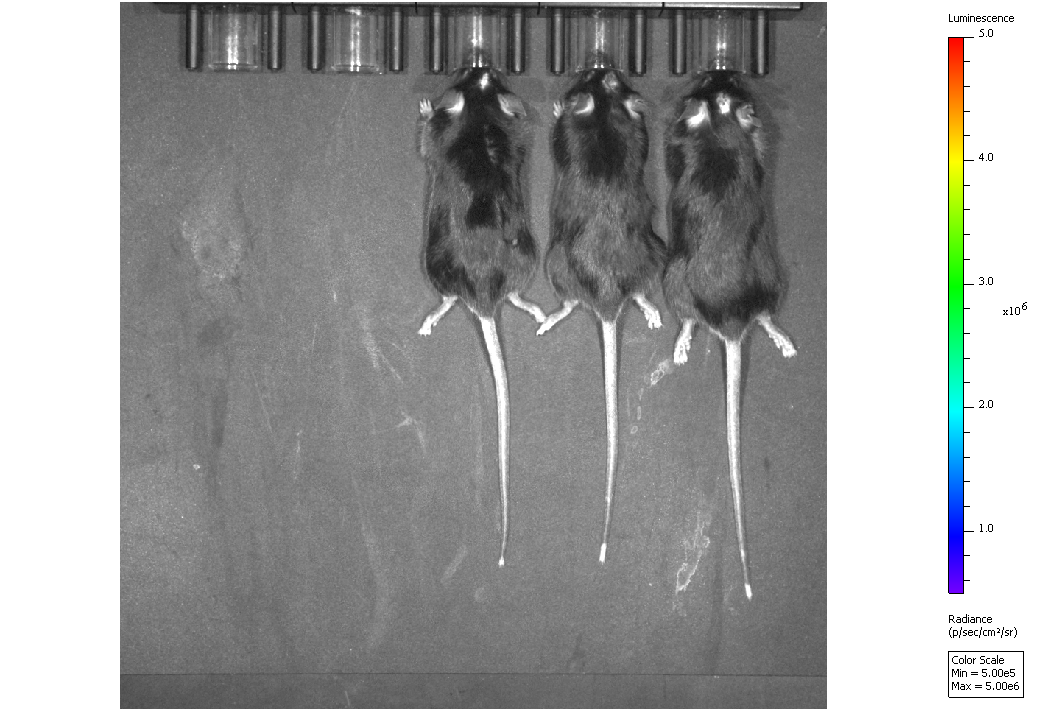
shP4HA1(day7)
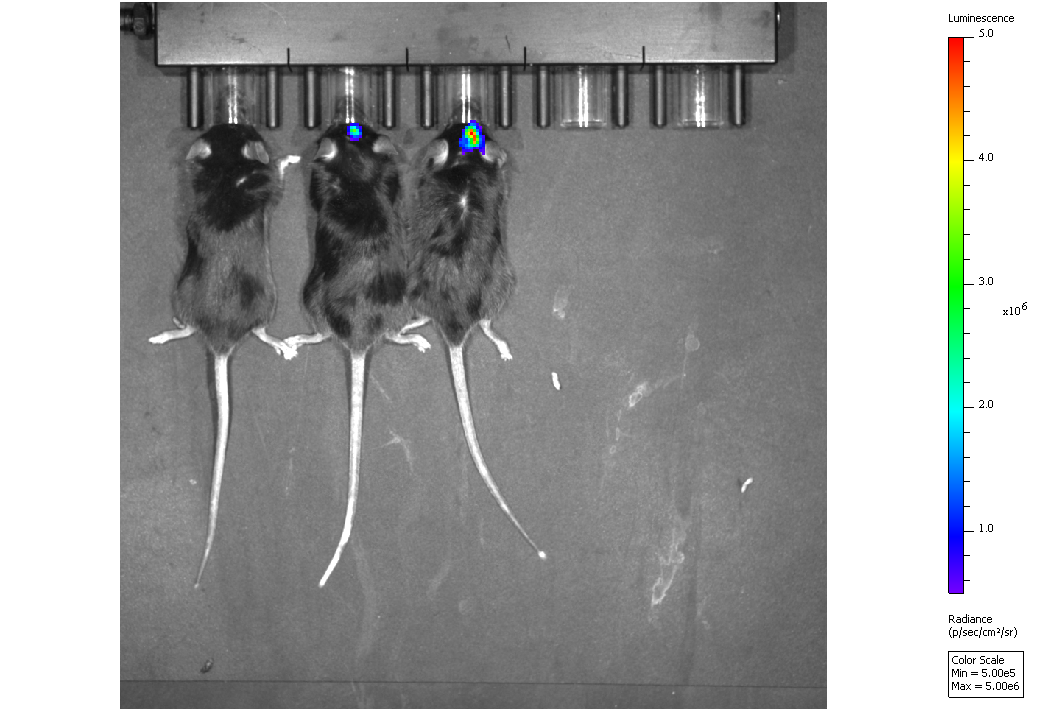
 shP4HA1(day 14)


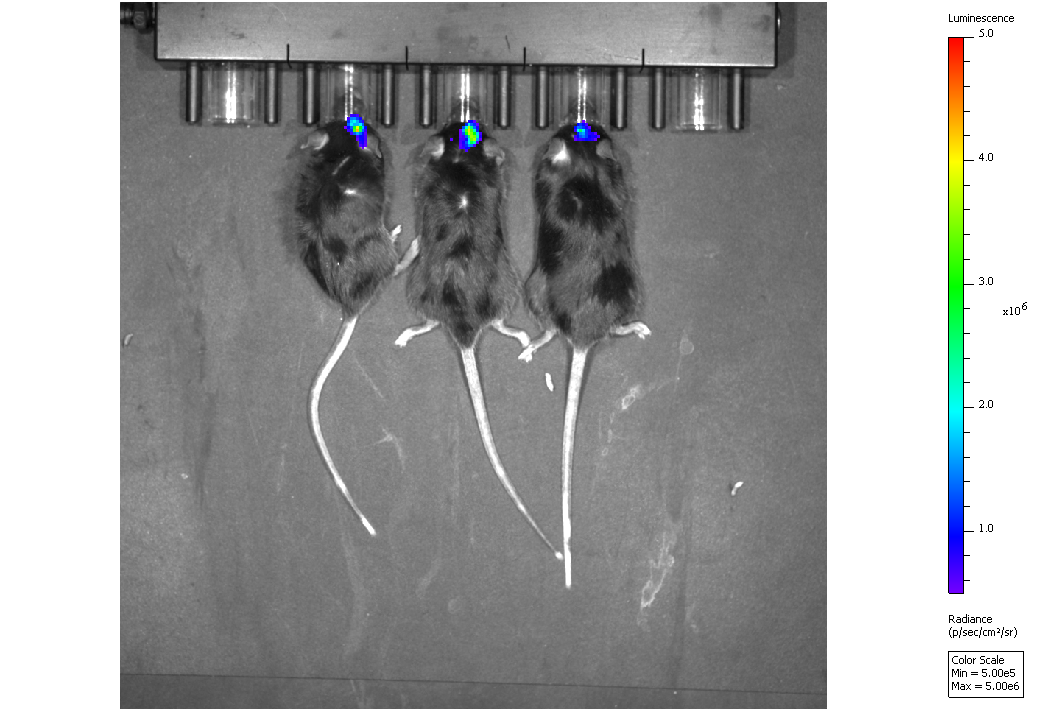
oeCtrl(day7)
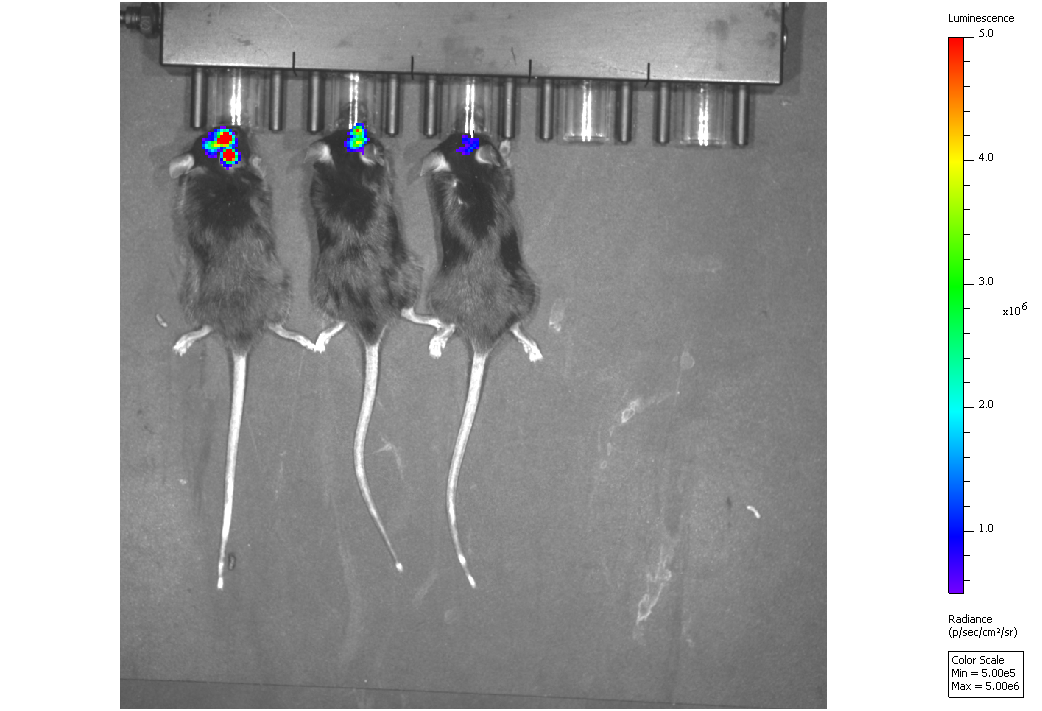
 oeCtrl(day14)
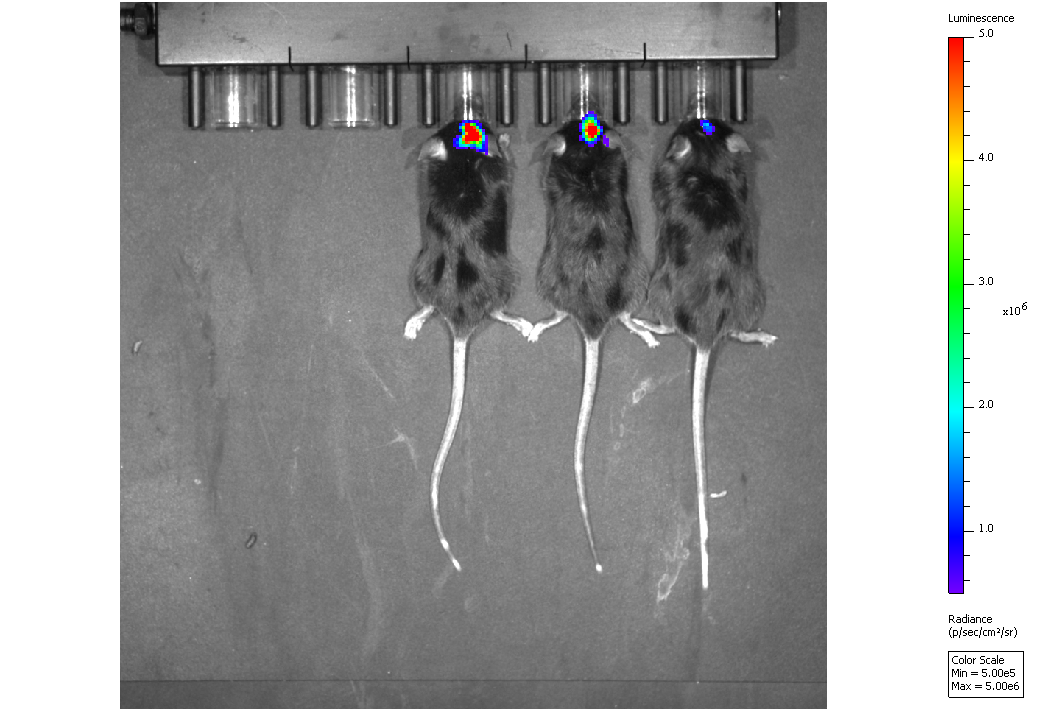
oeP4HA1(day 7)
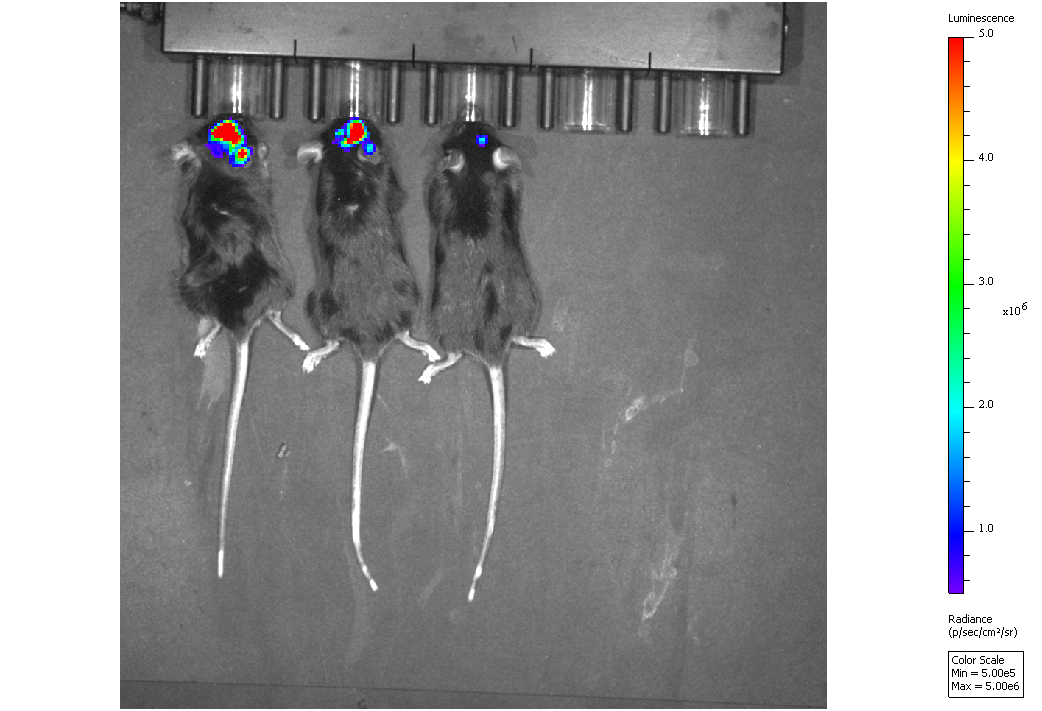
 oeP4HA1(day 14)

| Days after injection | shCtrl | | | shP4HA1 | | | oeCtrl | | | oeP4HA1 | | |
| --- | --- | --- | --- | --- | --- | --- | --- | --- | --- | --- | --- | --- |
| 7 days | 1.872 | 3.375 | 2.215 | 0.723 | 0.58 | 0.893 | 1.856 | 1.586 | 1.165 | 2.085 | 3.321 | 4.164 |
| 14 days | 4.194 | 5.178 | 4.788 | 1.632 | 1.56 | 1.983 | 4.872 | 3.676 | 3.288 | 4.587 | 8.808 | 7.706 |

Figure 6B

ROI(10*6) value represent tumor volume in IVIS.

Figure 6C

4 groups, 6 mice per group for overall survival calculation and weight calculation

Overall survival (days after injection)

| Days elapsed | shCtrl | shP4HA1 | oeCtrl | oeP4HA1 |
| --- | --- | --- | --- | --- |
| 23 | 0 | 0 | 0 | 1 |
| 24 | 0 | 0 | 0 | 0 |
| 27 | 0 | 0 | 0 | 1 |
| 30 | 1 | 0 | 1 | 1 |
| 32 | 1 | 0 | 1 | 1 |
| 36 | 1 | 0 | 0 | 1 |
| 37 | 0 | 0 | 1 | 0 |
| 39 | 1 | 0 | 0 | 1 |
| 41 | 0 | 1 | 0 |  |
| 42 | 0 | 1 | 1 |  |
| 43 | 1 | 0 | 1 |  |
| 44 | 1 | 1 | 1 |  |
| 46 |  | 0 |  |  |
| 47 |  | 1 |  |  |
| 48 |  | 1 |  |  |
| 50 |  | 0 |  |  |
| 51 |  | 1 |  |  |

Figure 6D

average mice weight(g)

| Days after injection | shCtrl | shP4HA1 | oeCtrl | oeP4HA1 |
| --- | --- | --- | --- | --- |
| 0 | 32 | 30.1 | 31 | 32 |
| 7 | 29.9 | 29.9 | 30 | 28 |
| 10 | 27.6 | 28.3 | 28.6 | 25.6 |
| 14 | 25.6 | 26 | 27.4 | 24.3 |
| 18 | 24.4 | 25.4 | 24.6 | 20.8 |
| 22 | 22.3 | 24.7 | 22.8 | 18.4 |
| 26 | 20.3 | 24.9 | 21.6 | 17.3 |
| 30 | 19 | 23.7 | 20.2 | 15.7 |
| 34 | 17.3 | 22.9 | 19.3 | 15 |
| 38 | 16.7 | 21.6 | 17.6 | 13.9 |
| 42 | 15.4 | 21 | 15.3 |  |
| 46 |  | 20.4 | 14.6 |  |
| 50 |  | 19.3 |  |  |
