# Supplementary figures and images for: P4HA1 Regulates CD31 via COL6A1 in the Transition of Glioblastoma Stem-Like Cells to Tumor Endothelioid Cells
Source: Front Oncol. 2022 Apr 13;12:836511. doi: 10.3389/fonc.2022.836511 (PMC9044633; doi:10.3389/fonc.2022.836511)

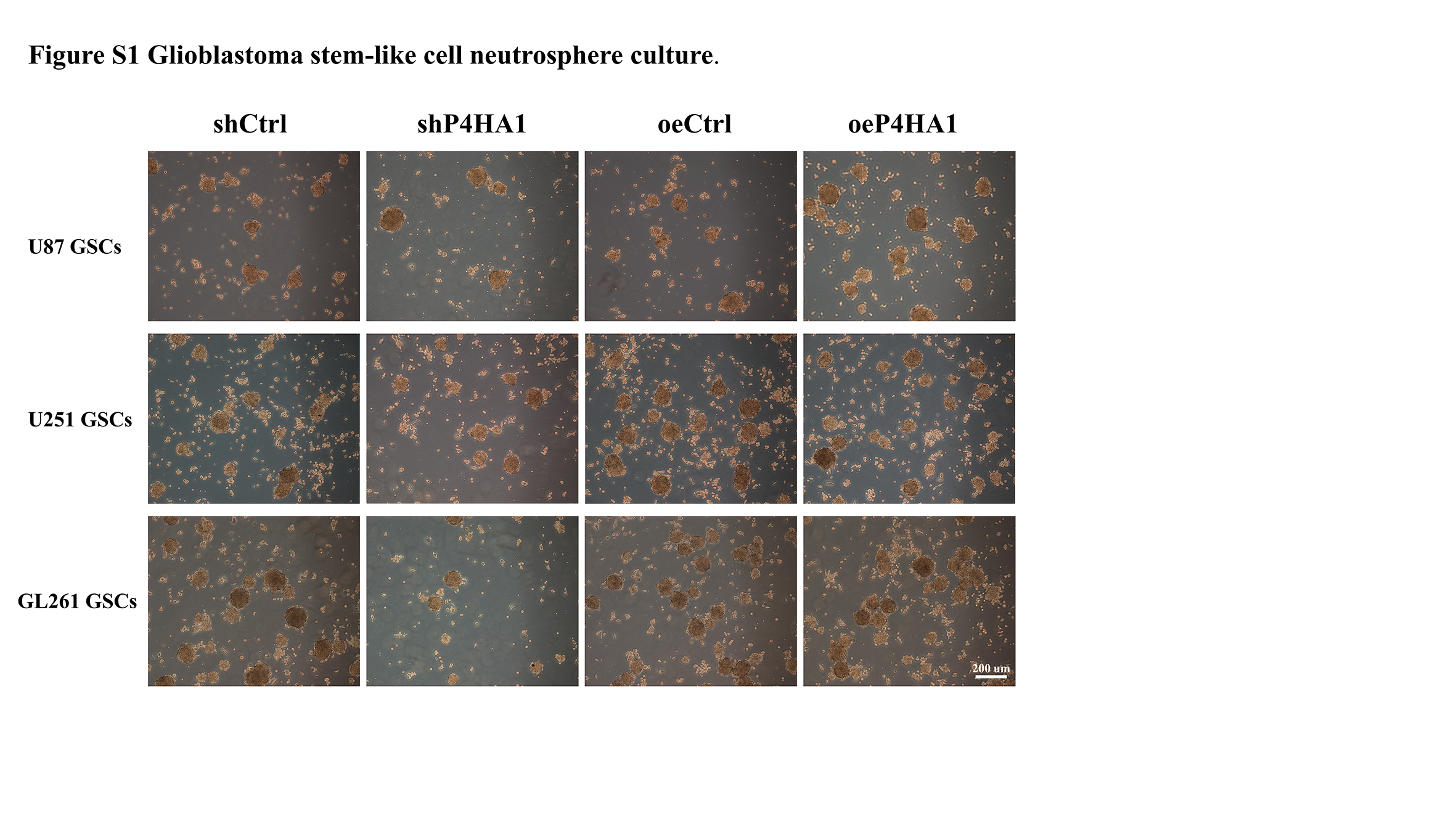

Supplement: Supplementary file 2 [file Image_1.tif]

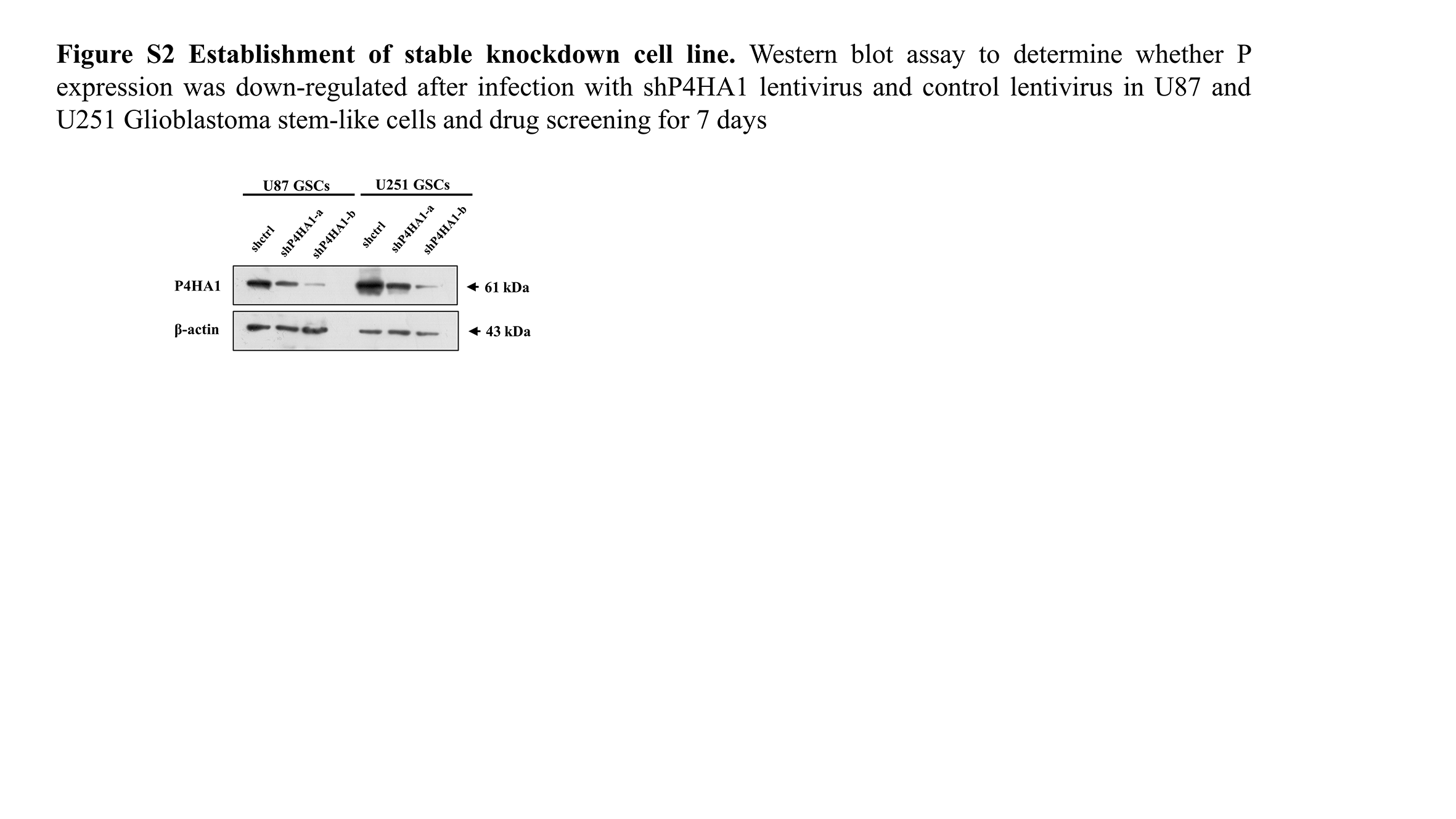

Supplement: Supplementary file 3 [file Image_2.tif]

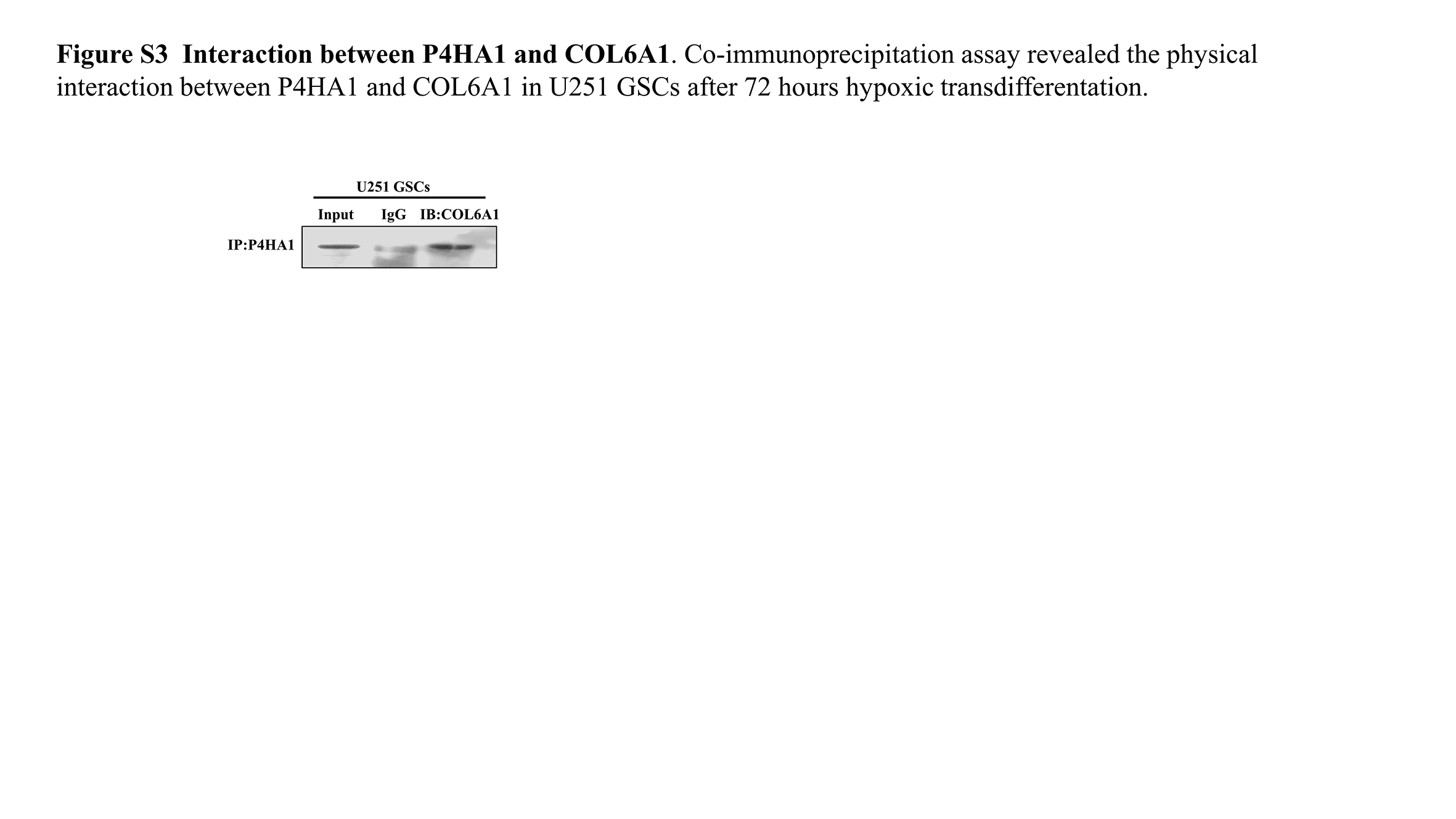

Supplement: Supplementary file 4 [file Image_3.tif]
